# Electrical stimulation inhibits Val-boroPro-induced pyroptosis in THP-1 macrophages via sirtuin3 activation to promote autophagy and inhibit ROS generation

**DOI:** 10.18632/aging.103038

**Published:** 2020-04-14

**Authors:** Lin Cong, Ziyu Gao, Yinghong Zheng, Ting Ye, Zitong Wang, Pengyu Wang, Manman Li, Bowen Dong, Wei Yang, Quanfeng Li, Shupei Qiao, Cao Wang, Yijun Shen, Hong Li, Weiming Tian, Liming Yang

**Affiliations:** 1 Department of Pathophysiology, Basic Medical Science, Harbin Medical University, Harbin 150081, China; 2 School of Life Science and Technology, Harbin Institute of Technology, Harbin 150006, China; 3 State Key Laboratory of Cardiovascular Disease, Fuwai Hospital, National Center for Cardiovascular Diseases, Beijing 100037, China

**Keywords:** electrical stimulation, pyroptosis, sirtuin3, ROS, macrophages

## Abstract

The incidence of atherosclerosis (AS), a major contributor to cardiovascular disease, is steadily rising along with an increasingly older population worldwide. Pyroptosis, a form of inflammatory programmed cell death, determines the release of pro-inflammatory mediators by endothelial cells, smooth muscle cells, and atheroma-associated macrophages and foam cells, thereby playing a critical role in AS progression. Canonical pyroptosis is mediated by inflammasome formation, activation of caspase-1, and maturation and release of proinflammatory cytokines. Electrical stimulation (ES) is a noninvasive, safe therapy that has been shown to alleviate symptoms in several health conditions. Here, we investigated the anti-inflammatory and anti-pyroptotic effects of ES in human THP-1 macrophages treated with the dipeptidyl peptidase inhibitor Val-boroPro (VbP). We found that ES downregulated NOD-like receptor family protein 3 (NLRP3) inflammasome, ASC, and caspase-1 expression and abrogated the release of Interleukin-1β (IL-1β) and Interleukin-18 (IL-18), indicating effective pyroptosis inhibition. These changes were paralleled by a reduction in reactive oxygen species (ROS) production, reversal of VbP-induced sirtuin3 (Sirt3) downregulation, deacetylation of ATG5, and induction of autophagy. These findings suggest that ES may be a viable strategy to counteract pyroptosis-mediated inflammation in AS by raising Sirt3 to promote autophagy and inhibit ROS generation.

## INTRODUCTION

Atherosclerosis (AS), a chronic inflammatory disease of the arterial wall characterized by gradual build-up of arterial plaque, is the main cause of cardiovascular disease (CVD) [1, 2]. Aging is a major, independent risk factor for AS [3]. Macrophages are main components of the inflammatory plaque and promote AS development across its different stages [4–6]. Accumulating data indicates that pyroptosis, a highly inflammatory form of programmed cell death usually triggered in immune cells upon infection by intracellular pathogens, occurs also in AS-associated macrophages and contributes importantly to plaque instability [7, 8]. Therefore, therapies aimed at inhibiting macrophage pyroptosis may reduce the morbidity and mortality associated with AS.

Programmed cell death (PCD) pathways, including apoptosis and necroptosis, are required for normal cell turnover and tissue homeostasis and their dysregulation has been implicated in aging and age-related diseases [9]. In AS-affected vessels, apoptosis induced by reactive oxygen species (ROS), nitric oxide, endotoxin, and oxidized LDL, among other factors, occurs in endothelial cells, smooth muscle cells, and macrophages and contributes to plaque vulnerability [10–12]. Pyroptosis, a novel pro-inflammatory PCD, is different from apoptosis and necrosis, which constitutes a distinct, pro-inflammatory form of PCD that is mediated by multiprotein complexes known as inflammasomes [13]. NOD-like receptor family protein 3 (NLRP3), the best characterized inflammasome type, is a crucial signaling node that controls caspase-1 mediated maturation of Interleukin-1β (IL-1β) and Interleukin-18 (IL-18) [14]. This is achieved by recruitment of the adapter protein ASC (apoptosis-associated speck-like protein containing a C-terminal caspase recruitment domain) to the NLRP3 complex, which results in cleavage of pro-caspase-1 into biologically active caspase-1 [15, 16]. It was shown that the NLRP3 inflammasome exacerbates atheroma formation, and its inhibition contributes to plaque stabilization [17]. Because ROS production promotes NLRP3 inflammasome formation, ROS inhibition can also be exploited to prevent pyroptosis in AS and other diseases [18, 19]. Sirtuin3 (Sirt3) is a member of the sirtuin family of proteins with NAD^+^-dependent protein deacetylase activity that regulate many important cellular processes, including transcription, metabolism, and oxidative stress responses [20, 21]. Interestingly, recent studies reported that Sirt3 activity increases autophagic flux in lipid-laden macrophages, preventing NLRP3 inflammasome activation and protecting cells from oxidative stress [22, 23]. Also of relevance for CVD, it has been shown that vascular Sirt3 expression in humans decreases with age, and forced Sirt3 expression greatly improves graft integration after vein-graft stenting in rats [24].

Endogenous bioelectric signals influence virtually every physiological process in the body. Since the proposal, about 100 years ago, of the injury-healing properties of endogenous electric fields, a large number of experimental studies have confirmed the influence of bioelectricity on pathophysiological responses of cells and tissues [25–27]. Increasingly adopted in Physical Therapy practice, electrical stimulation (ES) has shown to promote the healing and regeneration of skin, bone, muscle, and nervous tissue [28]. Different cells respond differently to electrical stimulation (ES), which may alter electrophysiological activity in electrogenic cells, or induce proliferation or differentiation in non-electrogenic cells [29]. ES significantly reduced the release of cytokines and chemokines within the ischemic penumbra [30]. Another study found that low-frequency ES attenuated muscle atrophy in a mouse model of chronic kidney disease by stimulating myogenesis and inducing delayed M2 polarization of infiltrating macrophages [31]. In addition, intraoperative ES was shown to decrease oxidative stress and induce autophagy in the hemidiaphragm of surgery patients undergoing mechanical ventilation [32]. In fact, ES has been used clinically to treat various health conditions, such as gastrointestinal and neurological diseases, but the specific therapeutic mechanisms are not entirely clear.

The present work explored whether ES can inhibit oxidative stress and reduce the production of ROS by up-regulating Sirt3 and enhancing autophagy, and then reducing the occurrence of VbP -induced pyroptosis in THP-1 macrophages.

## RESULTS

### VbP induces pyroptosis in THP-1 macrophages

**Figure 1:**
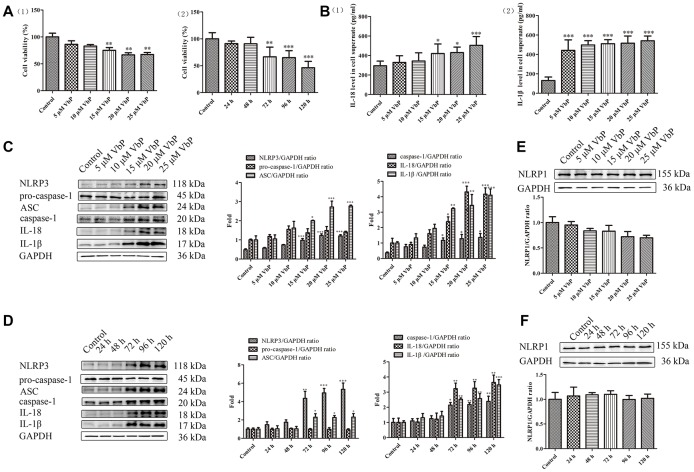
**VbP induces pyroptosis in THP-1 macrophages.** (**A**) Detection of cell viability (CCK-8 assay) after (1) 72 h exposure to different concentrations of VbP, and (2) exposure to 15 μM VbP for various times. (**B**) ELISA results showing secretion of IL-18 (1) and IL-1β (2) by macrophages treated for 72 h with different concentrations of VbP. (**C**) Dose-dependent expression of pyroptosis-related proteins in untreated (control) and VbP-treated macrophages (72 h exposure). (**D**) Time-dependent expression of pyroptosis-related proteins in untreated (control) and VbP-treated (15 μM) macrophages. (**E**) Dose-response analysis of NLRP1 inflammasome expression in VbP-treated macrophages (72 h exposure). (**F**) Time-response analysis of NLRP1 expression in macrophages exposed to 15 μM VbP. n = 3; *P<0.05, **P<0.01, and ***P<0.001 vs. control cells.

To determine the optimal pyroptosis induction conditions, cell viability was analyzed in VbP -treated, PMA-differentiated THP-1 macrophages using the CCK-8 assay. Dose- and time-response experiments showed that over a 72 h incubation time, cell viability was reduced significantly at VbP concentrations ≥15 μM (Figure 1A_(1)_). In turn, when 15 μM VbP were applied, cell survival decreased significantly at incubation times ≥72 h (Figure 1A_(2)_). Meanwhile, after incubation with 15 μM VbP for 72 h, ELISA results showed that IL-18 and IL-1β levels in cell culture supernatants increased significantly (Figure 1B). In parallel, western blot results from similar dose-response (Figure 1C) and time-response (Figure 1D) studies showed that 15 μM VbP exposure for 72 h induced a significant increase in the expression of NLRP3, ASC (a scaffold protein that links NLRP3 and pro-caspase-1 [33]), caspase-1, IL-18, and IL-1β. We also confirmed that the expression of NOD-like receptor family protein 1 (NLRP1), another inflammasome complex also involved in pyroptosis, did not change significantly under the above conditions (Figure 1E and 1F). Therefore, in subsequent experiments pyroptosis was elicited in THP-1 macrophages by applying 15 μM VbP during a 72 h exposure time.

### ES inhibits VbP-induced pyroptosis in THP-1 macrophages

**Figure 2:**
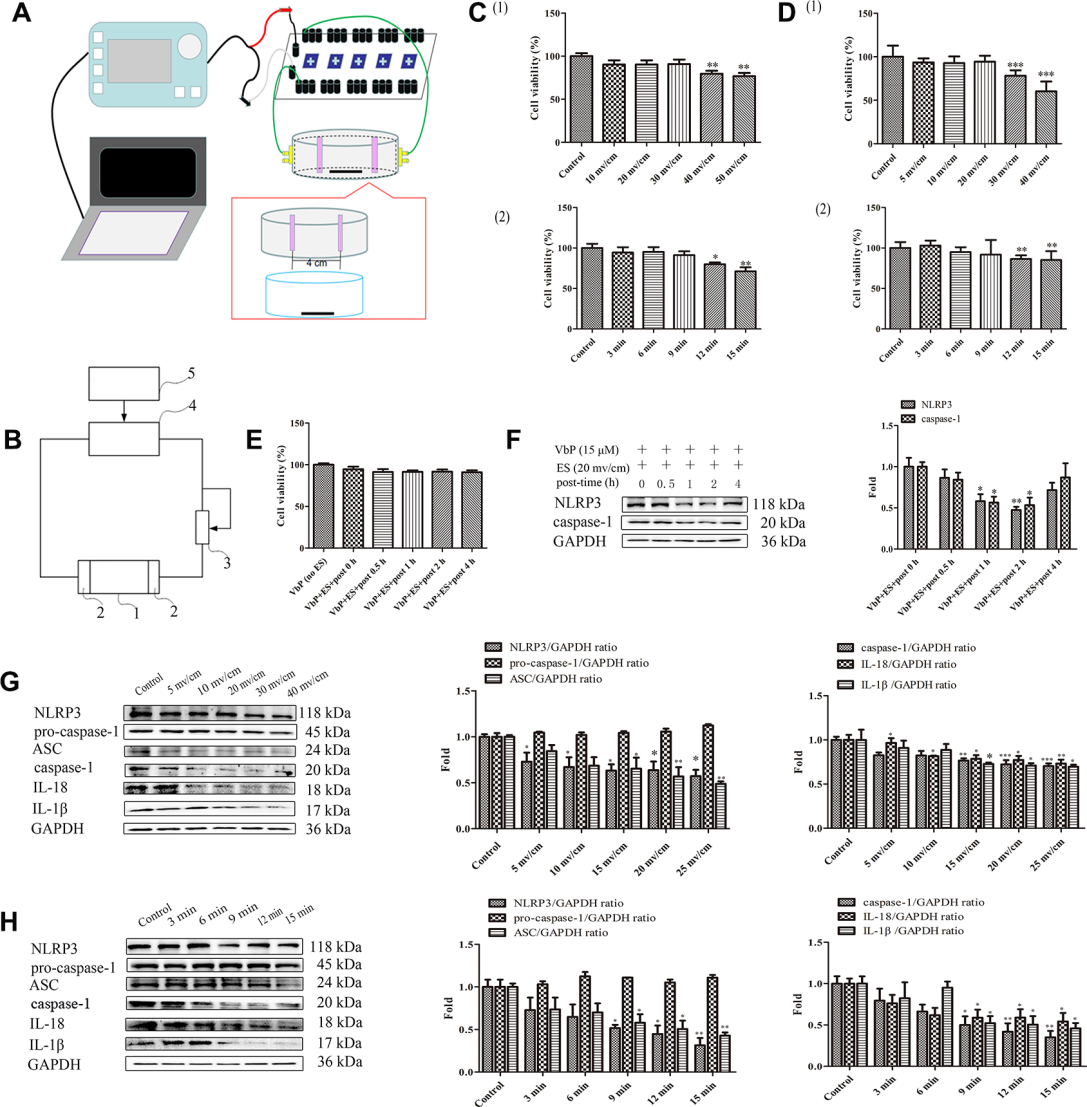
**ES inhibits VbP-induced pyroptosis in THP-1 macrophages.** (**A**) Pattern diagram of the ES device. (**B**) Circuit diagram of the ES device. (**C**) Viability assay results in control THP-1 macrophages after ES at (1) different voltages (9 min exposure) and (2) 30 mv/cm for various exposure times. (**D**) Viability assay results in macrophages exposed to VbP (15 μM, 72 h) and subsequently treated with ES at (1) different voltages (9 min exposure) and (2) 20 mv/cm for different exposure times. (**E**) Viability measurements at different time points after ES (20 mv/cm for 9 min) in macrophages treated with VbP (15 μM, 72 h). (**F**) Western blot analysis of NLRP3 and caspase-1 protein expression at different time points after ES. (**G**) Dose-response analysis of the expression of pyroptosis-related proteins in macrophages treated with ES (9 min exposure) after VbP treatment (15 μM, 72 h). (**H**) Time-dependent analysis of the expression of pyroptosis-related proteins in macrophages treated with ES (20 mv/cm) after VbP exposure (15 μM, 72 h). n =3; *P<0.05, **P<0.01, and ***P<0.001 vs. control cells.

Figure 2A shows the modules and Figure 2B the circuit diagram of the ES device. Optimal ES parameters (i.e. voltage and treatment times) were selected by running cell viability assays to ensure that ES treatment per se did not compromise cell survival. The data showed that the survival rate of untreated THP-1 macrophages decreased significantly when an ES ≥40 mv/cm was applied during 9 min (Figure 2C_(1)_). In subsequent time-response tests using 30 mv/cm ES, we determined that viability was significantly reduced in control cells when ES lasted ≥12 min (Figure 2C_(2)_). Similar experiments in macrophages treated with 15 μM VbP indicated that 20 mv/cm and 9 min exposure were the upper threshold limits above which cell viability decreased modestly (Figure 2D). To evaluate whether delayed cell death may occur after short-term ES, viability was examined in VbP-treated macrophages between 0 and 4 h following ES. Results showed that cell viability was unchanged up to 4 h after a 9 min, 20 mv/cm ES (Figure 2E). Meanwhile, western blot results indicated that the expression of pyroptosis associated NLRP3 and caspase-1 proteins in VbP-treated THP-1 macrophages declined significantly only after 1 to 2 h following a 9 min ES at 20 mv/cm (Figure 2F). Therefore, downstream analysis were performed after a resting period of 2 h (at 37 °C, 5% CO_2_) following ES. To conclude ES settings optimization, the influence of both voltage and ES exposure time on pyroptosis-related protein levels was evaluated by western blot. As shown in Figure 2G and 2H, an obvious decrease in all the proteins tested (except for pro-caspase 1) was evident in cells treated with VbP and then subjected to ES (9-min, 20 mv/cm). Accordingly, 20 mv/cm and 9 min exposure time were chosen as the optimal ES parameters to characterize the effects of ES on VbP-induced pyroptosis in THP-1 macrophages.

### ES inhibits caspase-1-dependent pyroptosis of THP-1 macrophage induced by VbP

**Figure 3:**
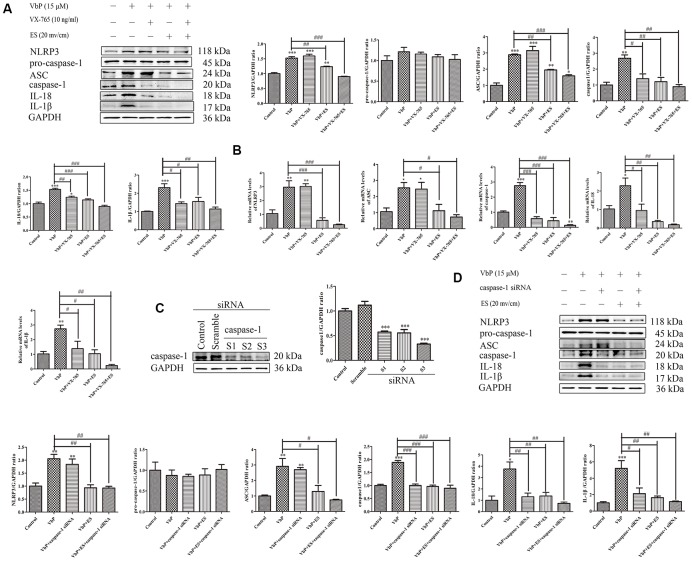
**ES inhibits caspase-1-dependent pyroptosis of THP-1 macrophage induced by vbp.** (**A**) Western blot analysis of the effect of caspase-1 inhibition on pyroptosis-related proteins. (**B**) RT-qPCR analysis of the effect of caspase-1 inhibition on pyroptosis-related mRNAs. (**C**) Representative western blot results showing downregulation of caspase-1 expression following siRNA-mediated caspase-1 knockdown. (**D**) Western blot analysis of the effect of caspase-1 knockdown on pyroptosis-related proteins. n = 3; *P<0.05, **P<0.01, and ***P<0.001 vs. control cells; ^#^P<0.05, ^##^P<0.01, and ^###^P<0.001 vs. VbP-treated cells.

Pyroptosis is triggered by caspase-1 after activated by various inflammasomes and results in lysis [34]. To confirm whether pyroptosis depends on caspase-1 activation in our experiments, we detected the expression of pyroptosis-related proteins by western blot after exposing PMA-differentiated THP-1 macrophages to VbP in the absence (control) or presence of the caspase-1 inhibitor VX-765. Results showed that VX-765 inhibited VbP-induced caspase-1, IL-18, and IL-1β expression without affecting NLRP3 nor ASC levels. Likewise, after VbP treatment and except for pro-caspase-1, the expression of all the above proteins was also reduced by ES (Figure 3A). Through RT-qPCR assays, we further determined that these treatments resulted in changes in mRNA expression that were consistent with protein analysis results (Figure 3B). To further examine the relationship between pyroptosis and caspase-1, we knocked down caspase-1 via siRNA (Figure 3C). In line with the above results, IL-18 and IL-1β expression was reduced, while NLRP3 and ASC levels were unaffected, in VbP -treated THP-1 macrophages following caspase-1 knockdown (Figure 3D). These data indicate that ES inhibits VbP-induced pyroptosis on THP-1 macrophages by inhibiting inflammasome formation, caspase-1 activation, and production of pro-inflammatory interleukins.

### ES decreases VbP -induced ROS generation in THP-1 macrophages

**Figure 4:**
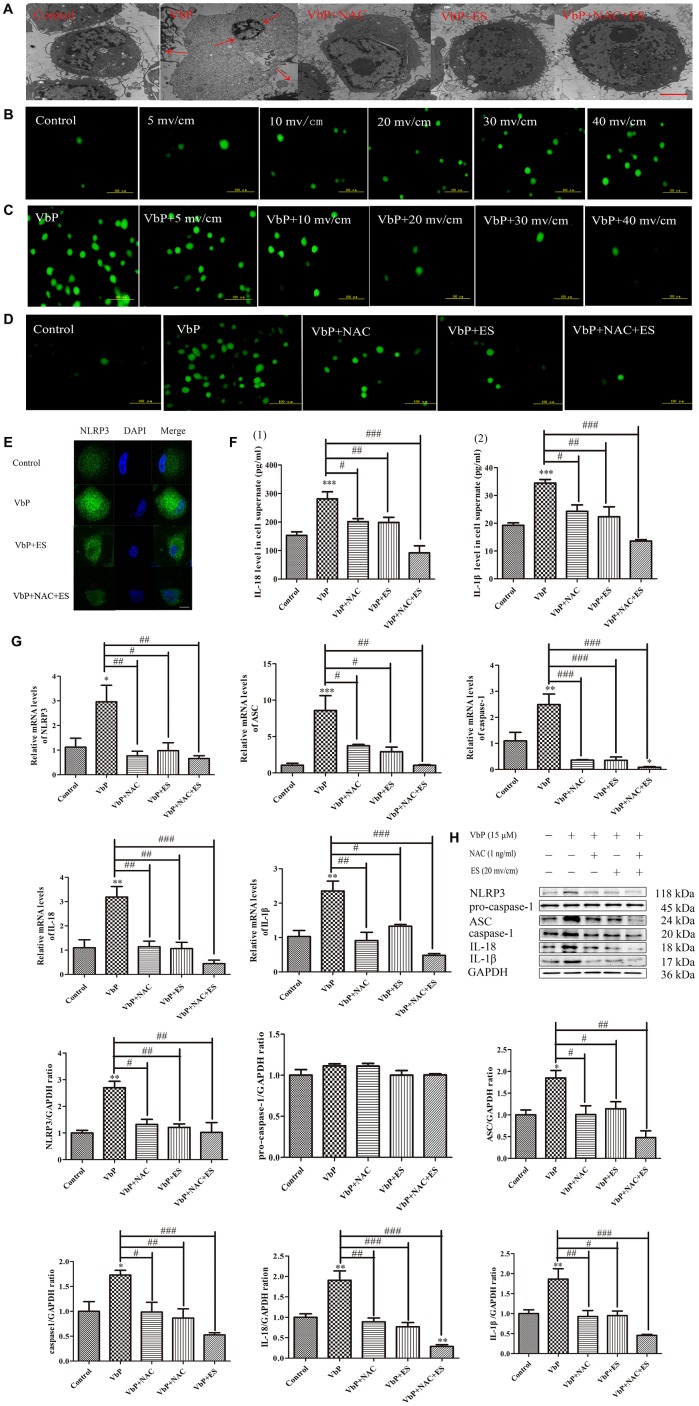
**ES inhibits ROS production in VbP-treated THP-1 macrophages.** (**A**) TEM analysis of ultrastructural alterations in THP-1 macrophages. Red arrows indicate cell swelling and nuclear pyknosis (scale bar: 10 μm). (**B**) DCFH-DA staining showing ROS generation in control macrophages following ES at different voltages (scale bar: 100 μm). (**C**) DCFH-DA staining showing ROS generation in VbP-treated macrophages following ES at various voltages (scale bar: 100 μm). (**D**) DCFH-DA staining showing ROS generation in VbP-treated macrophages exposed to NAC and ES (scale bar: 100 μm). (**E**) Effect of ES, alone or combined with NAC, on NLRP3 immunofluorescence in VbP-treated macrophages (scale bar: 20 μm). (**F**) ELISA analysis of the effects of NAC and ES on (1) IL-18 and (2) IL-1β secretion by THP-1 macrophages. (**G**) RT-qPCR analysis of the effects of NAC and ES on pyroptosis-related mRNAs. (**H**) Western blot analysis of the effects of NAC and ES on pyroptosis-related proteins. n =3; *P<0.05, **P<0.01, and ***P<0.001 vs. control cells; ^#^P<0.05, ^##^P<0.01, and ^###^P<0.001 vs. VbP-treated cells.

Research has shown that reactive oxygen species (ROS) facilitate activation of the NLRP3 inflammasome [35]. To assess ROS generation during VbP-induced pyroptosis, and whether ES counteracts this effect, N-acetylcysteine (NAC) was used to inhibit ROS production in THP-1 macrophages. Ultrastructural TEM analysis of VbP-treated cells showed that cell enlargement and nuclear shrinkage, i.e. two typical morphological changes in cells undergoing pyroptosis, were prevented by both co-application of NAC (1 mM) or subsequent ES, either alone or in combination (Figure 4A). As shown in Figure 4B, ROS production (assessed by 2-7-Dichlorofluorescin diacetate (DCFH-DA) staining) was stimulated in control THP-1 macrophages by raising the voltage on ES tests. Likewise, VbP exposure dramatically increased ROS production, although this effect was clearly reversed by subsequent exposure to ES (≥ 10 mv/cm) (Figure 4C) or concurrent NAC addition (Figure 4D). Besides, immunofluorescence results showed that VbP-induced NLRP3 expression decreased significantly after treatment with NAC, ES (20 mv/cm), or both (Figure 4E). Moreover, NAC exposure significantly attenuated IL-18 and IL-1β secretion (Figure 4F), and decreased the expression of pyroptosis-related mRNA transcripts (Figure 4G) and proteins (Figure 4H) in VbP-treated macrophages. These data indicate that VbP induces pyroptosis in THP-1 macrophages via ROS generation, which is effectively attenuated by ES.

### ES relieves pyroptosis of THP-1 macrophage by upregulating Sirt3 to activate autophagy and inhibit oxidative stress

**Figure 5:**
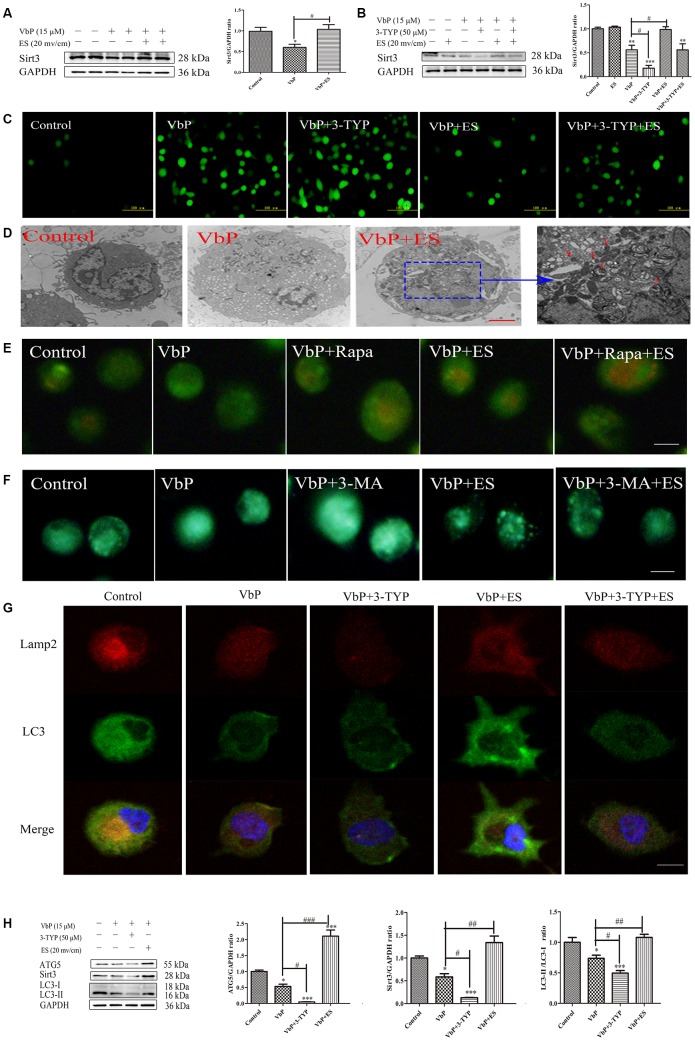
**ES rescues Sirt3 expression to induce autophagy in VbP-treated THP-1 macrophages.** (**A**) Western blot analysis of Sirt3 expression in macrophages treated with VbP and ES. (**B**) Analysis of the effects of ES and the Sirt3 inhibitor 3-TYP on Sirt3 expression. (**C**) DCFH-DA staining showing the effect of 3-TYP on ROS generation by macrophages (scale bar: 100 μm). (**D**) TEM images showing autophagosome assembly (red arrows) in VbP-treated macrophages exposed to ES (scale bar: 10 μm). (**E**) Visualization of AVOs by AO staining. The autophagy inducer Rapa (1 μM) was used as positive control. (**F**) DMC staining showing induction of autophagic vacuoles by ES in VbP-treated macrophages. This effect was blocked by pre-treatment with the autophagy inhibitor 3-MA (10 mM) (scale bar: 50 μm). (**G**) Immunofluorescence analysis of LC3 and Lamp2 expression indicating induction of autolysosome formation by ES and inhibition of this process by 3-TYP in VbP-treated macrophages (scale bar: 20 μm). (**H**) Western blotting analysis of the effects of ES and 3-TYP on ATG5, Sirt3, and LC3 expression in VbP-treated macrophages. n = 3; *P<0.05, **P<0.01, and ***P<0.001 vs. control cells; ^#^P<0.05, ^##^P<0.01, and ^###^P<0.001 vs. VbP-treated cells.

Most ROS production occurs in the mitochondria and is directly influenced by Sirt3, a NAD+-dependent deacetylase located mainly in this organelle. Since we showed that ES reduces VbP-induced ROS production in THP-1 macrophages, we asked whether Sirt3 upregulation may contribute to this effect. Western blots showed that Sirt3 levels decreased following VbP exposure, but fully recovered after subsequent ES (Figure 5A). ES had no effect on Sirt3 expression in untreated control cells, and pre-treatment with the Sirt3 inhibitor 3-TYP prevented ES-mediated Sirt3 upregulation in VbP-treated macrophages (Figure 5B). Meanwhile, DCFH-DA staining showed that 3-TYP exposure also attenuated the decrease in ROS levels mediated by ES in VbP-treated cells (Figure 5C). Since Sirt3 upregulation has been linked to induction of autophagy, we next assessed autophagosome formation by TEM [36]. This analysis revealed that ES promoted formation of autophagosomes with a distinct double membrane structure in VbP-treated macrophages (Figure 5D). Further evidence of active autophagy was provided by acridine orange (AO) staining, which indicated that ES also favored acidic vesicle organelles (AVOs) formation in those cells (Figure 5E). Meanwhile, monodansylcadaverine (MDC) staining showed diffuse labeling with minimal puncta in both control and VbP-treated cells. However, upon exposure of the latter to ES, numerous MDC-labeled autophagic vacuoles were evident, and this effect that was negated by pre-treatment with the autophagy inhibitor 3-methyladenine (3-MA) (Figure 5F). Further confirmation of Sirt3-mediated induction of autophagic fluxes following ES was obtained by evaluating autophagosome and autolysosome formation by immunofluorescence. Results showed that the expression of both microtubules associated protein 1 light chain 3-β (LC3), an autophagosome marker, and Lamp2, a marker of functional lysosomes, was decreased in VbP-treated macrophages, enhanced by subsequent ES, and abrogated by 3-TYP pre-treatment (Figure 5G). Furthermore, western blotting showed that the expression of LC3 and autophagy-related 5 (ATG5) was significantly increased by ES in VbP-treated cells, but decreased sharply after adding 3-TYP (Figure 5H). These findings indicate that pyroptosis inhibition by ES is linked to autophagy induction in macrophages.

**Figure 6:**
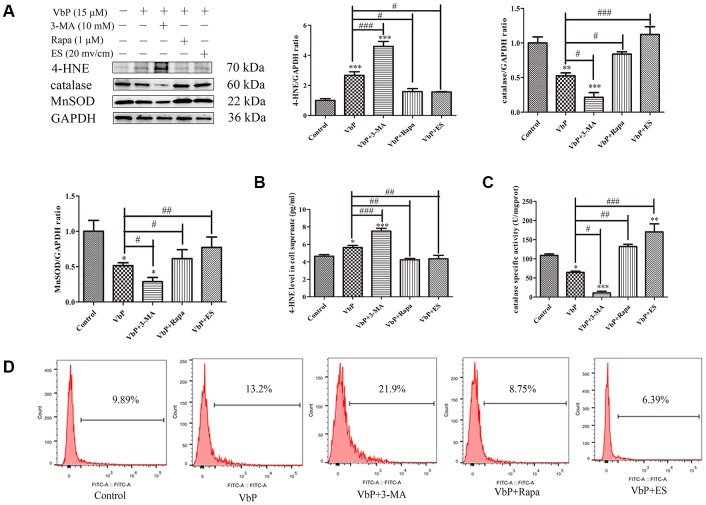
**ES prevents VbP-induced oxidative stress in THP-1 macrophages.** (**A**) Western blot analysis of the expression of 4-HNE adducts, catalase, and MnSOD. (**B**) ELISA determination of 4-HNE secretion. (**C**) Quantification of catalase activity (colorimetric detection). (**D**) Flow cytometry analysis of ROS generation in DCFH-DA labeled macrophages. n = 3; *P<0.05, **P<0.01, and ***P<0.001 vs. control cells; ^#^P<0.05, ^##^P<0.01, and ^###^P<0.001 vs. VbP-treated cells.

We further assessed whether ES could attenuate or reverse VbP-induced oxidative stress by analyzing the expression of 4-hydroxynonenal (4-HNE) protein adducts, indicative of lipid peroxidation, as well as two key antioxidant enzymes, i.e. catalase and MnSOD. As shown in Figure 6A, 4-HNE adduct levels were increased significantly by VbP exposure, further augmented by co-administration of 3-MA, and attenuated by pre-treatment with the autophagy inducer rapamycin (Rapa) and/or subsequent ES. On the other hand, both catalase and MnSOD expression was reduced by VbP, further decreased by co-treatment with 3-MA, and increased by co-incubation with Rapa or exposure to ES. In addition, ELISA showed that the expression of 4-HNE was increased in culture supernatants from VbP-treated cells, further augmented by 3-MA addition, and similarly attenuated by either Rapa or ES (Figure 6B). We also assessed catalase activity through a colorimetric assay, and detected changes in enzyme activity that reflected the corresponding protein expression changes described above (Figure 6C). Finally, ROS production measured by DC-FH-DA flow cytometry proved that ROS generation in VbP-treated THP-1 macrophages decreased after treatment with Rapa or ES, and increased instead after co-application of 3-MA (Figure 6D).

### ES promotes deacetylation of ATG5 by upregulating Sirt3

**Figure 7:**
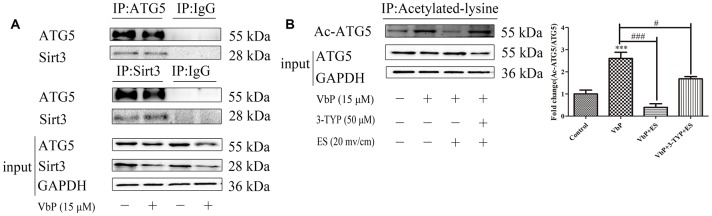
**ES induces Sirt3-mediated deacetylation of ATG5 in VbP-treated macrophages.** (**A**) Representative western blots of ATG5 expression following immunoprecipitation with an anti-Sirt3 antibody (top), Sirt3 expression following immunoprecipitation with an anti-ATG5 antibody (middle) and ATG5 and Sirt3 expression in total cell lysates (bottom). (**B**) Representative western blot and quantitative analysis of acetylated-ATG5 in macrophages treated with VbP, 3-TYP, and ES. n =3; ***P<0.001 vs. control cells; ^#^P<0.05 and ^###^P<0.001 vs. VbP-treated cells.

Recent research showed that some sirtuin family members, including Sirt1 and Sirt3, can bind and deacetylate ATG5, allowing phagophore elongation and maturation into a double-membrane autophagosome [22, 37, 38]. To examine if the Sirt3/ATG5 interaction is promoted by ES in our macrophage model of VbP-induced pyroptosis, we first combined MitoTracker staining and immunofluorescence and found that both Sirt3 and ATG5 were predominately expressed in mitochondria (Supplementary Figure 1). Meanwhile, co-immunoprecipitation experiments confirmed that Sirt3 levels were decreased in VbP-treated THP-1

macrophages, however, expression of the Sirt3-ATG5 complex did not change appreciably in response to VbP (Figure 7A). Based on these observations, we next sought to understand whether ATG5 was a substrate for Sirt3. By using an antibody directed against acetyl-lysine residues to immunoprecipitate ATG5, we demonstrated that ATG5 acetylation increased in response to VbP, decreased after subsequent ES, and was also reduced, but remained above basal levels, after pre-treatment with 3-TYP (Figure 7B). These data suggest that ES promotes Sirt3-mediated deacetylation of ATG5, leading to autophagosome formation in VbP-treated macrophages.

### ES inhibits VbP-induced pyroptosis in macrophage-derived foam cells

**Figure 8:**
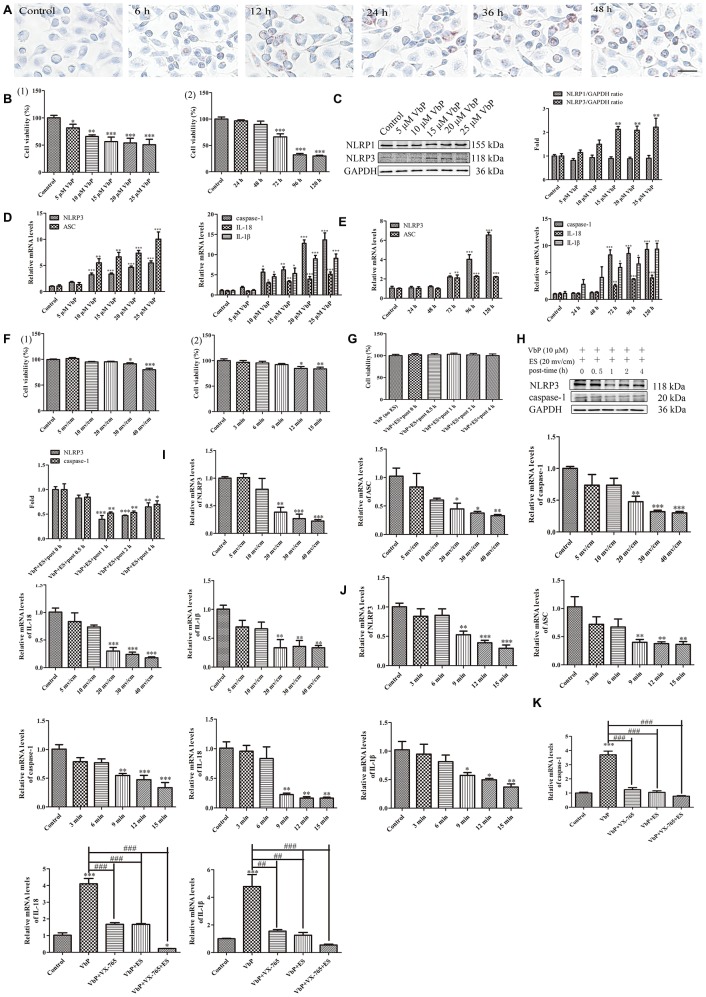
**ES inhibits VbP-induced pyroptosis in foam cells.** (**A**) Representative images of Oil Red O-stained THP-1 macrophages following incubation with ox-LDL for 6, 12, 24, and 48 h (scale bar: 50 μm). (**B**) Detection of foam cell viability by CCK8 assays: (1) Dose-response assay results (72 h incubation); (2) Time-response assay results (5 μM VbP). (**C**) Detection of NLRP1 and NLRP3 expression by western blotting in foam cells treated with different concentrations of VbP (72 h exposure). (**D**) Dose-response and (**E**) time-response RT-qPCR analysis of pyroptosis-related mRNAs in foam cells treated with different concentrations of VbP (72 h) or 10 μM VbP for up to 120 h. (**F**) Viability measurements in VbP-treated foam cells exposed to ES at (1) different voltages (9 min exposure) and (2) 20 mv/cm for various exposure times. (**G**) Viability measurements at different time points after ES (20 mv/cm for 9 min) in foam cells treated with VbP (10 μM for 72 h). (**H**) Analysis of NLRP3 and caspase-1 expression by western blotting at different time points after 9 min, 20 mv/cm ES. (**I**) Dose-response and (**J**) time-response RT-qPCR analysis of pyroptosis-related mRNAs in foam cells exposed to ES. (**K**) RT-qPCR analysis showing the effects of VbP, VX-765, and ES on caspase-1, IL-18, and IL-1β mRNA levels in foam cells. n = 3; *P<0.05, **P<0.01, and ***P<0.001 vs. control cells; ^##^P<0.01 and ^###^P<0.001 vs. VbP-treated cells.

Since lipid-laden macrophages, i.e. foam cells, are main contributors to plaque deposition and inflammation in AS [39], we asked whether ES may also prevent VbP-induced pyroptosis in foam cells. Ox-LDL was used to induce foam cell formation in THP-1 macrophage cultures. Oil Red O assays showed that after 24 h treatment macrophages began to phagocytose ox-LDL, indicating foam cell development (Figure 8A). Next, optimal pyroptosis induction conditions were explored using the CCK8 assay in foam cells obtained after 24 h incubation with ox-LDL. A significant inhibition of cell viability was noted after 72 h treatment with VbP concentrations ≥5 μM (Figure 8B_(1)_) and after 72 h exposure with 5 μM VbP (Figure 8B_(2)_). Through western blotting, we next verified the expression of NLRP1 and NLRP3 after 72 h incubation with different VbP concentrations. Results showed that the expression of NLRP1 did not change, while NLRP3 increased evidently when ≥15 μΜ VbP was applied (Figure 8C). We also examined the expression of pyroptosis-related mRNAs by RT-qPCR, and found that NLRP3, ASC, caspase-1, IL-18, and IL-1β all increased distinctly after 72 h exposure to ≥10 μM VbP (Figure 8D and 8E). Accordingly, we chose 10 μM and a 72-h treatment time to evaluate VbP-induced pyroptosis in foam cells.

Subsequently, dose- and time-response experiments were conducted to assess the effect of ES on the viability of VbP-treated foam cells. Results showed that following 9-min exposure, modest but significant reductions in viability occurred at voltages ≥30 mv/cm, while at a fixed voltage of 20 mv/cm, viability was not affected during exposures lasting ≤9 min (Figure 8F). To evaluate whether delayed onset of cell death may occur during recovery from ES, CCK8 assays were performed in VbP-treated foam cells between 0 and 4 h following ES. No significant changes in cell viability were detected up to 4 h after 9 min ES at 20 mv/cm (Figure 8G). Meanwhile, under the same ES conditions western blot results indicated that the expression of the pyroptosis-associated proteins NLRP3 and caspase-1 began to decline 1 h post-ES (Figure 8H). Through further dose- and time-response experiments, RT-qPCR assays showed that 20 mv/cm and 9 min exposure were the lowest threshold settings causing significant reductions in NLRP3, ASC, caspase-1, IL-18, and IL-1β mRNA levels in VbP-treated foam cells (Figure 8I and 8J). Therefore, the above ES settings were found to be optimal to inhibit VbP-induced pyroptosis in THP-1 macrophage-derived foam cells. Applying these settings, RT-qPCR showed that caspase-1, IL-18, and IL-1β mRNA levels were dramatically increased by VbP, and greatly reduced by both co-treatment with VX-765 and subsequent ES (Figure 8K). These findings demonstrate that ES also inhibits VbP-mediated pyroptosis in foam cells.

## DISCUSSION

**Figure 9:**
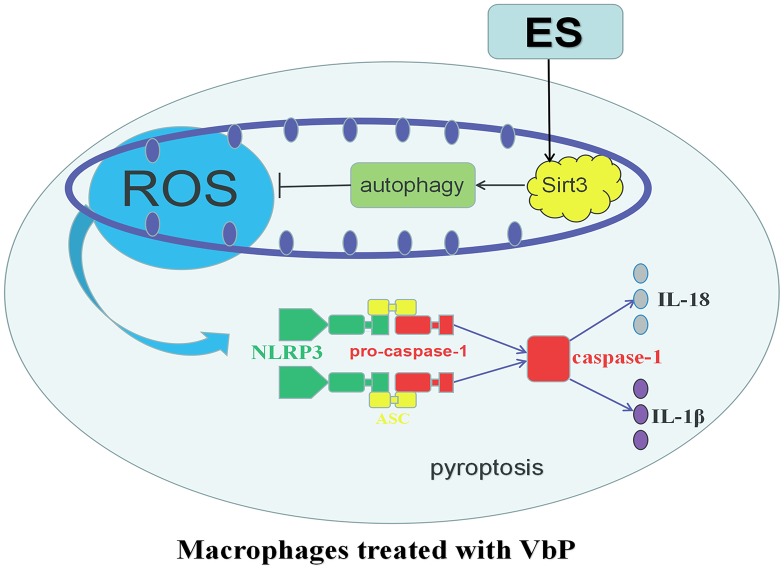
**Schematic representation of the effects of ES on VbP-treated THP-1 macrophages.** VbP exposure induces ROS production, which promotes NLRP3 inflammasome formation leading to activation of caspase-1. Activated caspase-1 triggers pyroptosis through membrane pore formation, DNA fragmentation, and release of mature IL-1β and IL-18. The ensuing sterile inflammatory response contributes in turn to the progression of AS. Meanwhile, ES normalizes Sirt3 expression and promotes autophagy in VbP-treated macrophages. This reduces ROS production and prevents oxidative stress, which inhibits NLRP3 inflammasome formation and prevents pyroptosis.

After ES of THP-1 macrophages treated with VbP, the increase of Sirt3 expression accelerated the occurrence of autophagy and inhibited the production of ROS effectively, which inhibited the expression of NLRP3, resulting in ASC unable to recruit pro-caspase-1 and induce its own cleavage to form mature caspase-1, and the decrease of IL-18 and IL-1β expression (Figure 9).

Macrophages participate in the initiation and development of AS by secreting various factors that drive local inflammation, atheroma formation, and plaque rupture and thrombosis, and all these processes are exacerbated by their conversion into cholesterol-laden foam cells [40, 41]. Mounting evidence suggests that, macrophages in advanced AS lesions undergo pyroptosis a lytic, pro-inflammatory form of programmed cell death, which contributes importantly to necrotic core formation and plaque instability [42]. Accordingly, therapeutic approaches that exploit intrinsic cell death mechanisms in macrophages and macrophage-derived foam cells represent a promising strategy for the prevention and treatment of AS [43]. Attending to this need, our study employed a human macrophage model of pyroptosis to support the potential applicability of ES to effectively inhibit macrophage pyroptosis and reduce AS morbidity and mortality.

It is well known that NLRP1 and NLRP3 inflammasomes mediate the production of various cytokines in inflammatory disorders [44]. Inhibition of the highly related cytosolic serine proteases Dpp8/9 by VbP has been demonstrated to trigger pyroptosis selectively in monocytes and macrophages, and reported to activate the inflammasome sensor protein NLRP1b in mouse RAW264.7 macrophages [45]. In this study we observed that VbP exposure did not alter the expression of NLRP1 in human THP-1 macrophages, while the expression of NLRP3 increased obviously. At present, both classical and non-classical pyroptosis pathways have been identified. The main steps of the classical (canonical) pathway include activation of both inflammatory bodies and caspase-1, an enzyme that promotes the maturation of IL-1β and IL-18 precursors. This form of pyroptosis has been described in monocytes, macrophages, and dendritic cells [46]. We showed that this mechanism underlies VbP-mediated pyroptosis in THP-1 macrophages, as both ES and caspase-1 inhibition (with VX-765) or knockdown (via siRNA) significantly attenuated the increase in IL-18 and IL-1β induced by VbP. In contrast, unlike ES, VX-765 was unable to reduce VbP-induced NLRP3 and ASC expression, thus confirming that NLRP3 and ASC are located upstream of caspase-1.

Ever since inflammasomes, the macromolecular complexes responsible for caspase-1 activation, emerged as key regulators of immune and inflammatory responses, modulation of inflammasome activity has become an important therapeutic goal [47]. ROS generation is considered an important factor in the induction of the NLRP3 inflammasome [48]. Our data showed that ROS levels in THP-1 macrophages were enhanced by VbP exposure, and this increase, as well as pyroptosis, were inhibited independently or in combination by NAC and ES. In line with results of a previous study, we noted that ES elicited a dose-dependent stimulation of ROS production in untreated THP-1 macrophages [49]. However, our data also showed that progressively higher voltages reduced instead ROS generation in VbP-treated cells. These data suggest that that ES may have opposite effects on ROS generation in macrophages depending on their physiological state. Research has shown that ES induces structural and functional changes in mitochondria from different cells [50, 51]. Unlike sirtuin1 (Sirt1), which shows context-dependent shuttling between the nucleus and the cytosol, Sirt3 is expressed exclusively in mitochondria [52]. Excessive production of mitochondrial ROS impairs mitochondrial integrity and homeostasis and promotes activation of the NLRP3 inflammasome, which contributes to vascular inflammation and dysfunction [53, 54]. Sirt3 activity inhibits ROS generation by deacetylation of major mitochondrial antioxidant enzymes, namely isocitrate dehydrogenase 2, superoxide dismutase, and glutathione peroxidase [55]. Moreover, Sirt3 can promote or inhibit autophagy by interacting with different partners, such as AMPK, Foxo3a, and SOD2, and its deficiency was shown to negatively impact the development of myocardial ischemia-reperfusion injury [56]. For instance, a recent animal study showed that Sirt3 deficiency exacerbated diabetic cardiomyopathy, while Sirt3 overexpression promoted mitochondrial homeostasis and cardiomyocyte survival through induction of mitophagy and autophagy via Sirt3-Foxo3A-Parkin signaling [57]. Meanwhile, Liu et al. recently showed that reduced Sirt3 levels paralleled autophagy inhibition and NLRP3 activation in peripheral blood monocytes from obese humans and in THP-1 cells exposed to palmitate. In addition, the same authors showed that autophagosome formation was positively influenced by Sirt3-dependent deacetylation of ATG5 [22]. Here we showed that Sirt3 expression was reduced in THP-1 macrophages exposed to VbP. However, subsequent ES restored Sirt3 levels, stimulated the expression of LC3 and Lamp2, and decreased ATG5 acetylation. These data clearly indicated that ES inhibited pyroptosis by promoting autophagy in cultured macrophages. We also showed that ROS generation and lipid peroxidation (4-HNE production), i.e. two processes stimulated by VbP, were further increased by addition of the autophagy inhibitor 3-MA, and decreased significantly after co-addition of the autophagy agonist Rapa. These data are consistent with evidence that autophagy prevents pyroptosis by inhibiting the production of ROS [58].

In summary, our study suggests that ES can effectively inhibit VbP-induced, NLRP3/caspase-1-mediated pyroptosis in THP-1 macrophages by inducing autophagy through upregulation of Sirt3, inhibition of ROS production, and deacetylation of ATG5. These findings suggest that ES may be a useful approach to inhibit inflammation caused by pyroptosis of arterial macrophage/foam cells, potentially reducing AS- and age-related vascular dysfunction. Nevertheless, our work has several limitations, since it only involved in vitro experiments, and focused only on caspase-1-dependent pyroptosis without examining potential Sirt3- and caspase-1 independent mechanisms of autophagy regulation. By binding and activating caspase-11/4/5, intracellular lipopolysaccharide can shear and activate the gasdermin D protein, isolate the active GSDMD-N end, form gasdermin channel in the cell membrane, and induce cell scorching, which is called nonclassical pathway of pyroptosis [59]. In this paper, it is not clear whether there is a non classical approach to pyroptosis. In addition, we don’t clear whether there is any Sirt3-independent mechanism for autophagy regulation. Others’ research clarifies that berberine associated photodynamic therapy promotes autophagy via ROS generation [60]. In addition, mitochondrial E3 ubiquitin ligase 1 promotes autophagy flux [61]. These autophagy mentioned in these articles is Sirt3-independent and may also exist in this study. It is unclear whether these molecules play a role in regulating autophagy in this project, which needs further research.

## MATERIALS AND METHODS

### Reagents

Val-boroPro (Talabostat mesylate; PT-100; Cat. B3941) was purchased from ApexBio (Houston, TX, USA). Agarose was purchased from Vivantis (CA, USA). N-acetyl cysteine (NAC), 3-Methyladenine (3-MA), acridine orange, and monodansylcadaverine (MDC) were purchased from Sigma Chemical Co. (St. Louis, MO, USA). MitoTracker was from Beyotime (Shanghai, China). Rapamycin was purchased from Selleckchem (Houston, TX, USA). 3-TYP was purchased from MCE (Monmouth Junction, NJ, USA). VX-765 was purchased from AdooQ Bioscience (Irvine, CA, US). Antibodies against ATG5, Sirt3, NLRP3, caspase-1, IL-1β, MnSOD, and catalase were purchased from Cell Signaling Technology (Beverly, MA, USA). Other antibodies included NLRP1, 4-HNE, and pro-caspase-1, from Abcam (Cambridge, UK), Lamp2, from Santa Cruz Biotechnology (Santa Cruz, CA, USA), IL-18, from Affinity Biosciences Inc. (Cincinnati, OH, USA), ASC, from Omnimabs Inc. (Irvine, CA, USA), LC3B, from Sigma Chemical Co., and GAPDH, from Yeasen Biotech. (Shanghai, China). Horseradish peroxidase-conjugated secondary antibodies were purchased from ZSGB-BIO (Beijing, China).

### Cell culture and experimental treatments

Human THP-1 monocytes (American Type Culture Collection, Manassas, VA, USA) were cultured in complete medium consisting of RPMI 1640 medium (HyClone, Logan, UT, USA) supplemented with 10% fetal bovine serum (FBS; HyClone). The cells were maintained at 37 °C, 5% CO_2_ and the medium was replaced every 2–3 days. To induce differentiation into macrophages, aliquots of THP-1 monocytes (1.0 × 10^5^ cells/ml) were added dropwise onto cover glasses in complete medium containing 100 ng/ml phorbol-12-myristate-13-acetate (PMA; EMD Biosciences, La Jolla, CA, USA). After two hours, 2 ml of complete medium were added to the dishes and incubation was prolonged for an additional 72 h. For downstream experiments, differentiated THP-1 macrophages were treated with 15 μM VbP for 72 h (unless indicated otherwise) and drugs and inhibitors, i.e. NAC (1 mM), 3-MA (10 mM), Rapa (1 μM), 3-TYP (50 μM), and VX-765 (10 ng/ml) were applied 1 h before ES. None of these agents exhibited any obvious cytotoxicity. To derive foam cells, PMA-differentiated THP-1 macrophages were incubated with 50 μg/ml of oxidized-low-density lipoprotein (ox-LDL, Yiyuan Biotechnologies, Guangzhou, China) in RPMI 1640 medium with 0.3% bovine serum albumin (BSA) for 24 h.

### ES system

The ES system was provided by the Harbin Institute of Technology (Harbin, China; utility patent applications on file). The system (Figure 2A and 2B) included: 1. *Cell culture system*, used to support living cells adhered to a cover glass in a 4-ml volume of RPMI 1640 medium; 2. *Electrode*. The salt bridge comprised a carbon plate and a plastic hollow pipe. The carbon plate was glued to the conductive copper foil adhesive tape with electrically conductive adhesive. One end of the plastic hollow pipe was fixed outside the carbon board with glass glue, then the pipe was injected with agar gel, and its free end inserted into the bottom of the cell culture dish; 3. *Resistance plate*, which allows to regulate electrical resistance through an adjustment knob; 4. *Electric signal generator*, which allows to adjust the voltage signal; 5. *Electric signal control unit,* which allows regulation of the output voltage signal by adjusting frequency, amplitude, bias, duty cycle, and waveform. The device is equipped adjustable voltage regulation and voltage detection circuits.

### Electrical stimulation (ES) protocol

After preparation of a fresh agarose gel salt bridge (0.01 mg/ml agarose), 4 ml RPMI 1640 medium was added to the ES device. After device debugging, the cover glass supporting THP-1 macrophages was clamped into the ES device. After ES (9 min at 20 mv/cm, unless indicated otherwise), the cells were returned to the incubator for an additional 2 h before examination.

### Cell viability assay

Cell survival rate was evaluated by the Cell Counting Kit 8 (CCK-8) assay (Beyotime Biotechnology, Jiangsu, China). THP-1 macrophages were seeded onto glass coverslips within 6-well culture plates, exposed to experimental treatments, and carefully washed with PBS. Then, 1 ml of medium containing CCK-8 (9:1, vol/vol) was added to each well. After incubation for 30 min at 37°C in the dark, each cell culture was transferred to 6 wells of a 96-well plate, into which 100 μL of medium containing CCK-8 was further added. Absorption (450 nm) was measured with a microplate reader (Varian Australia Pty Ltd., Australia). Data are expressed as the average of six wells for each treatment group.

### Enzyme linked immunosorbent assay (ELISA)

After treatments, culture supernatants from THP-1 macrophages were collected and soluble IL-18, IL-1β, and 4-HNE levels were measured by ELISA kits (Elabscience Biotechnology Co. Ltd., Wuhan, China) following the manufacturer’s protocols.

### Transmission electron microscopy (TEM)

After treatments, the cells were harvested by centrifugation and processed for TEM analysis on a JEM-1220 device (JEOL, Tokyo, Japan).

### Immunofluorescence

THP-1 cells were treated with ES, returned to the incubator for 2 h, washed with PBS, fixed with 4% paraformaldehyde for 30 min, and permeabilized with 1% Triton X-100 for 20 min at room temperature. Cells were then washed with PBS, blocked with 3% BSA, and incubated with NLRP3, Lamp2, LC3, Sirt3, or ATG5 antibodies (1: 200) overnight at 4 °C. The cells were then washed with PBS and incubated with fluorescently-labeled secondary antibodies at 37 °C for 1 h in the dark. DAPI staining was used to visualize cell nuclei. Cell fluorescence was observed on a laser scanning confocal microscope (LSM Meta, Carl Zeiss, Germany).

### Detection of reactive oxygen species (ROS)

ROS production was analyzed by measuring 2′-7′-dichloroflorescein diacetate (DCFH-DA) fluorescence. After ES treatment, the cells were washed twice with PBS and incubated with 20 μM DCFH-DA diluted in serum-free medium for 30 min at 37 °C in the dark. After PBS washing, the cells were analyzed via fluorescence spectrophotometry (Varian Australia Pty) or flow cytometry (FACSCalibur; BD Biosciences) using excitation and emission wavelengths of 488 nm and ~525 nm, respectively.

### Monodansylcadaverine (MDC), acridine orange, (AO) and MitoTracker stainings

MDC and AO stainings were used to visualize autophagic vacuoles and AVOs, respectively. After treatments, cells were incubated with 50 μM MDC or 5 μg/ml acridine orange for 30 min at 37 °C in the dark. To visualize mitochondria, after Sirt3 or ATG5 immunolabeling MitoTracker was applied at 37 °C for 30 min in the dark. After washing twice with PBS, the cells were observed under a fluorescence microscope (Olympus IX81; Japan).

### Western blot analysis

After treatments, the cells were lysed on an ice bath in RIPA buffer containing a protease inhibitor (PMSF). After quantification and denaturation, equal amounts of protein samples were electrophoresed in SDS-polyacrylamide gel and transferred onto PVDF membranes (Millipore, Schwalbach, Germany). The membranes were blocked for 1.5 h at room temperature with 5% dried skimmed milk in Tris-buffered saline with 0.05% Tween 20, and probed with specific primary antibodies at 4 °C overnight with slight agitation. Horseradish peroxidase (HRP)-conjugated secondary antibodies were next applied for 1.5 h at room temperature. Immunoreactivity was visualized by chemiluminescence using a ChemiDocTM MP Imaging System (Universal Hood III, Bio-Rad Laboratories, Inc., USA). Protein bands were quantified using Quantity One software analysis (Bio-Rad Laboratories, Hercules, CA, USA) and normalized to GAPDH.

### Quantitative PCR (RT-qPCR)

Total RNA was extracted using Trizol reagent (Invitrogen, Carlsbad, CA, USA) and reverse-transcribed using the RT Easy II First Strand cDNA Synthesis Kit (FORGENE, Sichuan, China). Then, 1 μL of cDNA was amplified in a Real-Time PCR Easy (SYBR Green I) (FORGENE, Sichuan, China) on an ABI 7900HT Sequence Detection system (ABI Applied Biosystems, Foster City, CA, USA). The following primers were used: NLRP3, forward 5’-CACCTGTTGTGCAATCTGAAG-3’ and reverse, 5’-GCAAGATCCTGACAACATGC-3’; ASC, forward 5’-AGGCCTGCACTTTATAGACC-3’ and reverse 5’-GCTGGTGTGAAACTGAAGAG-3’; caspase-1, forward 5’-CCTTAATATGCAAGACTCTCAAGGA-3’ and reverse 5’-TAAGCTGGGTTGTCCTGCACT-3’; IL-1β, forward 5’-TACCTGTCCTGCGTGTTGAA-3’ and reverse 5’-TCTTTGGGTAATTTTTGGGATCT-3’; IL-18, forward 5’-TGCATCAACTTTGTGGCAAT-3’ and reverse 5’-ATAGAGGCCGATTTCCTTGG-3’. Gene expression values were normalized against those of GAPDH, forward 5’-AAGGTCGGAGTCAACGGATTT-3’ and reverse 5’-AGATGATGACCCTTTTGGCTC-3’. Fold induction was calculated using the levels of expression of each gene at time 0 (untreated, control cells).

### Transfection of small interfering RNA (siRNA)

Knockdown of caspase-1 in THP-1 macrophages was performed according to the manufacturer’s protocols using siRNA oligonucleotides targeting the cDNA sequence of human caspase-1 (General Biosystems Inc., Anhui, China). To this end, 10 μL of siRNA (20 μM) was mixed with 100 μL Opti-MEM media (Invitrogen Life Technologies). Separately, 10 μL of X-tremeGene Transfection Reagent (Roche, Basel, Switzerland) was mixed with 100 μL Opti-MEM. The two samples were combined, and 20 min later the mixture was added into individual THP-1 macrophage cultures on a 35-mm dish containing fresh medium, and incubated for 6 h at 37 °C in the dark. Medium was then changed to antibiotic-free, FBS-containing RPMI 1640 medium for 48 h at 37 °C in the dark. Caspase-1 siRNA Duplexes S1 (sense: CCUGUGAUGUGGAGGAAAUTT, anti sense: AUUUCCUCCACAUCAXAGGTT), S2 (sense: UGGAAGACUCAUUGAACAUTT, anti sense: AUGUUCAAUGAGUCUUCCATT), S3 (sense: GAAGACUCAUUGAACAUAUTT; and anti sense: AUAUGUUCAAUGAGUCUUCTT) were used. A siRNA with an irrelevant nucleotide sequence was used as negative control.

### Catalase activity detection

Catalase activity was measured using a catalase Colorimetric Assay Kit (Elabscience Biotechnology Co., Ltd., Wuhan, China), according to manufacturer’s instructions.

### Immunoprecipitation

THP-1 macrophages were lysed with IP lysate and protein samples mixed with Sirt3 or ATG5 antibodies (2 μg) and 20 μL of 50% protein A/G-agarose bead slurry (Pierce Biotechnology, Rockford, IL, USA) overnight at 4 °C with gentle rotation. Immune complexes were washed 5 times with lysis buffer [50 mmol/l Tris (pH 7.4), 1% Triton X-100, 0.5% Nonidet P-40, 150 mmol/l NaCl, protease inhibitor (Calbiochem), and 10% (vol/vol) glycerol]. Samples were boiled in 2× sample buffer and then subjected to SDS-PAGE. The specificity of antibodies used for immunoprecipitation was routinely validated by running negative controls using non-immune IgG under the same conditions. For analysis of acetylated ATG5 in THP-1 cells, 1 mg of protein lysate was directly immunoprecipitated with an acetyl-lysine antibody and subjected to western blotting for Sirt3 and ATG5.

### Oil red O (ORO) staining

Staining with ORO (Sigma, St. Louis, MO, USA) was used to evaluate foam cell formation. Differentiated macrophages (80% confluence) were incubated with ox-LDL (50 μg/ml) in complete medium. At different time points, the cells were fixed with 4% paraformaldehyde and dehydrated with 60% isopropanol for 2 min. Next, neutral lipids were stained using a filtered 0.3% ORO solution for 10 min at room temperature. After rinsing with 60% isopropanol and ddH_2_O, hematoxylin was applied over 1 min to counterstain cell nuclei. ORO-stained foam cells were observed by light microscopy.

### Statistical analysis

All experiments were repeated at least three times independently. The data were analyzed using one-way analysis of variance (ANOVA) and student’s t-test. Results are presented as the mean ± standard deviation (SD). *P*<0.05 was considered significant.

## Supplementary Material

Supplementary Figure 1
