# Supplementary material for: Electrical stimulation inhibits Val-boroPro-induced pyroptosis in THP-1 macrophages via sirtuin3 activation to promote autophagy and inhibit ROS generation
Source: Aging (Albany NY). 2020 Apr 14;12(7):6415–35. doi: 10.18632/aging.103038 (PMC7185124; doi:10.18632/aging.103038)
Supplement: Supplementary Figure 1 [file aging-12-103038-s001..pdf]

SUPPLEMENTARY FIGURE

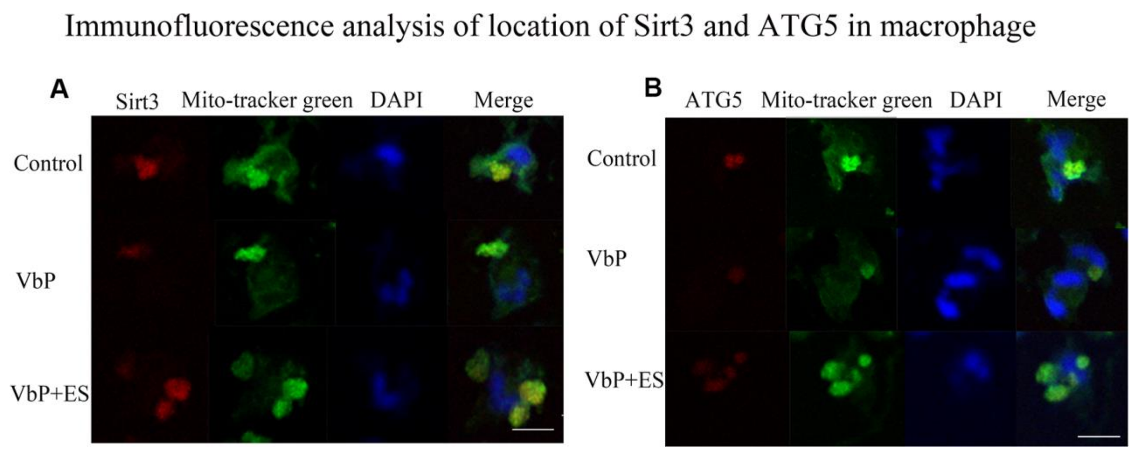

Supplementary Figure 1. Mitochondrial localization of Sirt3 and ATG5. (**A**) Sirt3 immunofluorescence (red) and MitoTracker staining (green) in THP-1 macrophages (Scale bar: 20 μm). (**B**) ATG5 immunofluorescence (red) and MitoTracker staining (green) in THP-1 macrophages (Scale bar: 20 μm).
